# Supplementary material for: Neuroblastoma Molecular Risk-Stratification of DNA Copy Number and ALK Genotyping via Cell-Free Circulating Tumor DNA Profiling
Source: Cancers (Basel). 2021 Jul 5;13(13):3365. doi: 10.3390/cancers13133365 (PMC8267662; doi:10.3390/cancers13133365)
Supplement: Supplementary file 1 [file cancers-13-03365-s001.zip › cancers-1251576-supplementary.pdf]

cancers

Supplementary Materials: Neuroblastoma Molecular Risk-Stratification of DNA Copy Number and *ALK* Genotyping via Cell-Free Circulating Tumor DNA Profiling

Smadar Kahana-Edwin, Lucy E. Cain, Geoffrey McCowage, Artur Darmanian, Dale Wright, Anna Mullins, Federica Saletta and Jonathan Karpelowsky

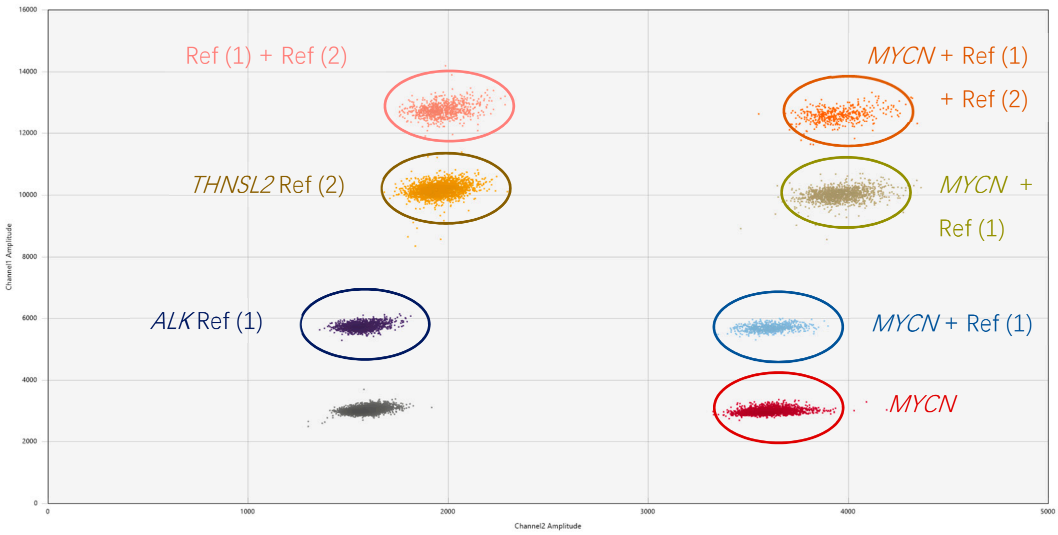

Figure S1. Simultaneous detection of the three different targets by ddPCR. Eight different clusters shown in a 2-D plot, represent different combinations of the detected targets (one negative cluster, two single positives, four double positives, and one triple positive). The capacity to separate discrete populations by levels of fluorescence enables multiplexing of three different targets.

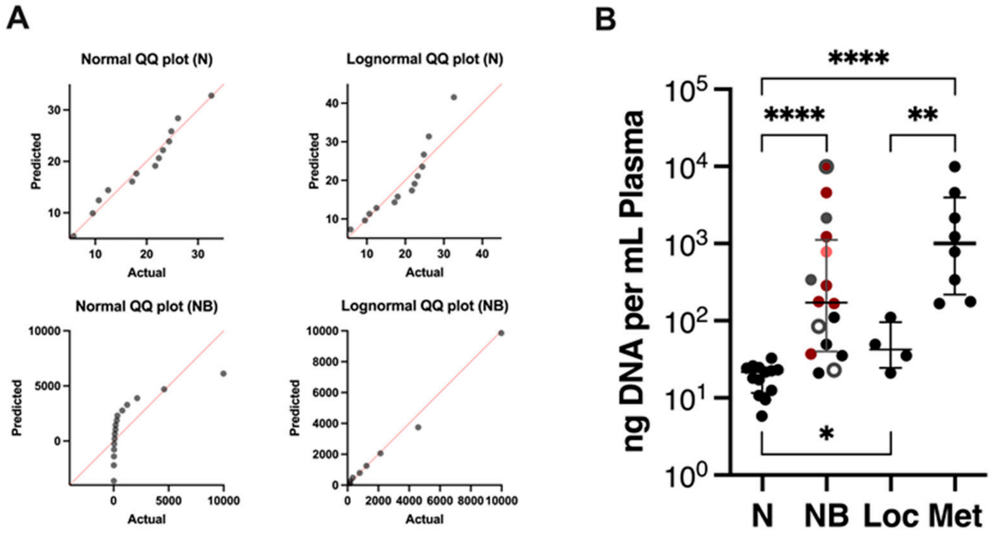

Figure S2. cfDNA distribution (**A**) and amount (**B**) from individuals diagnosed with neuroblastoma. (**A**) QQ plots for normal and lognormal distributions of cfDNA obtained from otherwise healthy patients in remission (N, top graphs) and active neuroblastoma (NB, bottom graphs) showing a normal distribution of cfDNA in remission samples and lognormal distribution in samples from patients with an active disease. This observation might reflect the nature of ctDNA origination: whereas cfDNA released from normal hematopoietic cells located in the circulation, ctDNA release into the bloodstream relays on additional factors (e.g. tumor vascularity). (**B**) Scatter plots presenting median values with the interquartile range. Loc – localized disease, Met – metastatic disease. NB samples in which SCA detected using SNP array colored grey / red / pink. NB sample in which

MYCN copy change detected with SNP array and ddPCR are colored according to: MNA – red, *MYCN* gain – pink. NB samples in which *ALK* mutations detected with ddPCR – hollow grey. P-values for a two-tailed Mann-Whitney test (distribution-free analysis) comparing between different disease states presented in asterisks (* – <0.05, ** – <0.01, **** – <0.0001).

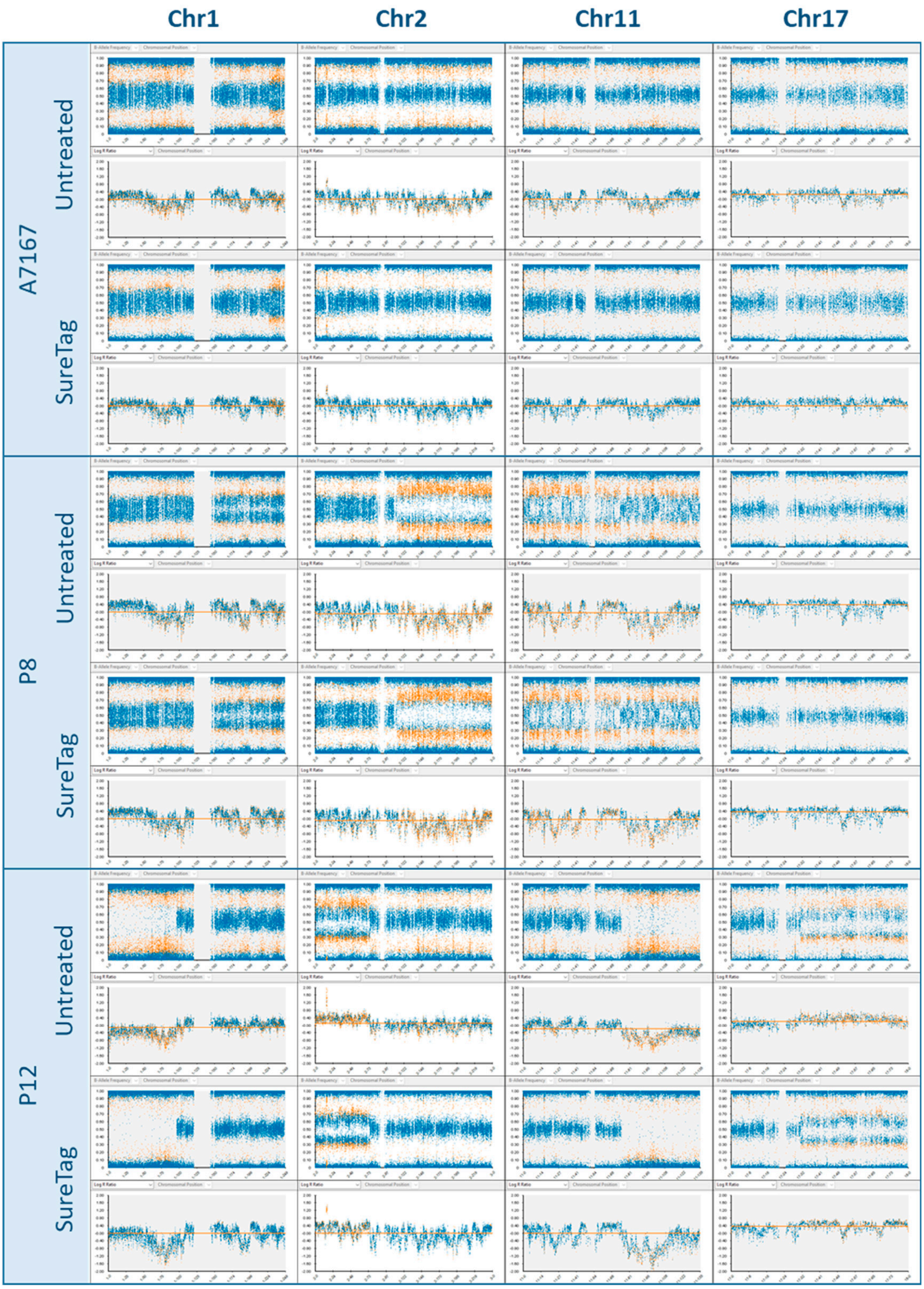

Figure S3. Enrichment of large cfDNA fragment does not improve the identification of variants in SNP array. Whole chromosomes 1, 2, 11, and 17 view of SNP array data of untreated and enriched cfDNA with the SureTag columns. BlueFuse Multi Analysis Software v4.5 B-allele frequency (top) and log R ratio (bottom) output profiles of cfDNA presented. Analysis of treated cfDNA does not reduced the noise observed with the untreated cfDNA.

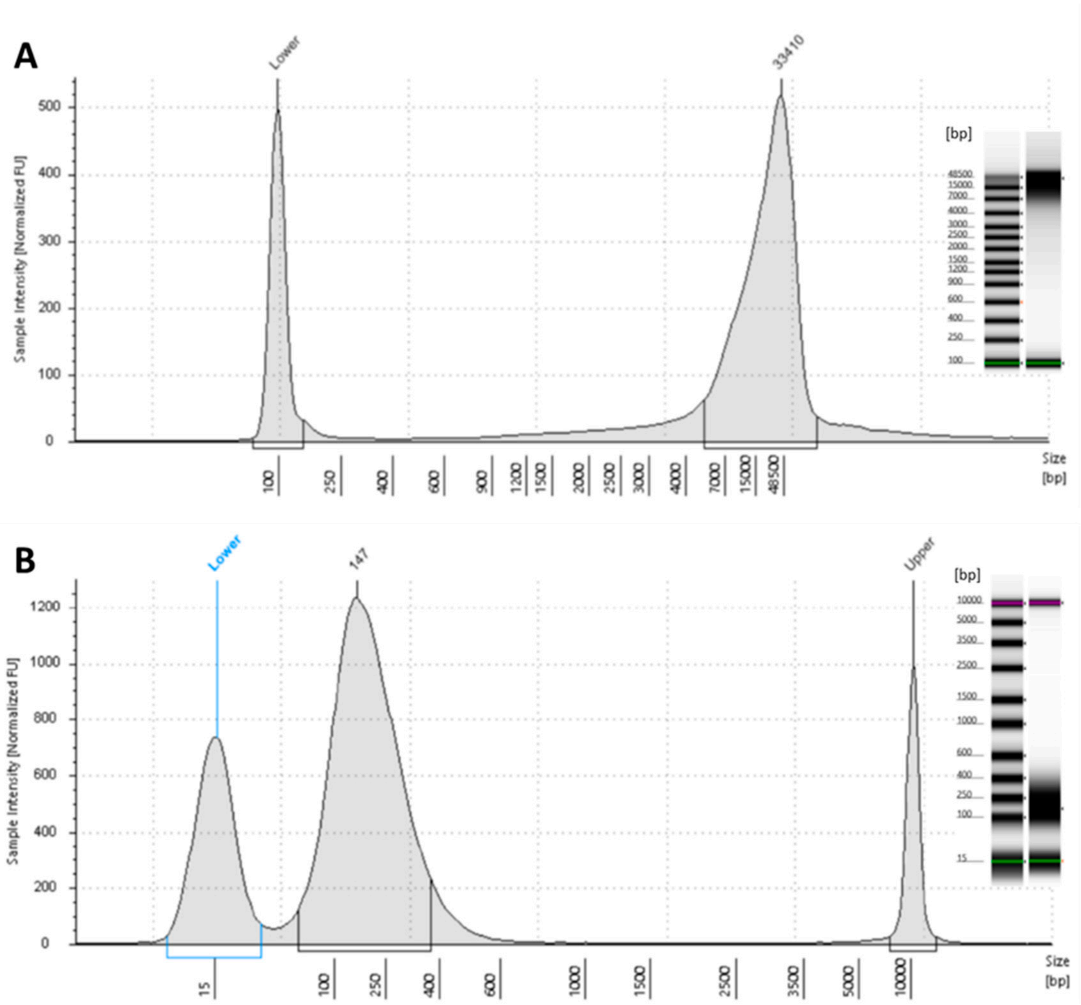

Figure S4. Constitutional DNA size-distribution used for cfDNA SNP array titration. Matched constitutional DNA was subjected to DNA fragmentation using Covaris Evolution sonicator. Electropherogram and gel images (smaller pictures) for DNA size and distribution were evaluated using TapeStation. (**A**) Pre-shearing DNA run on Genomic DNA ScreenTape® and (**B**) Post-shearing DNA run on High Sensitivity D5000 ScreenTape®.

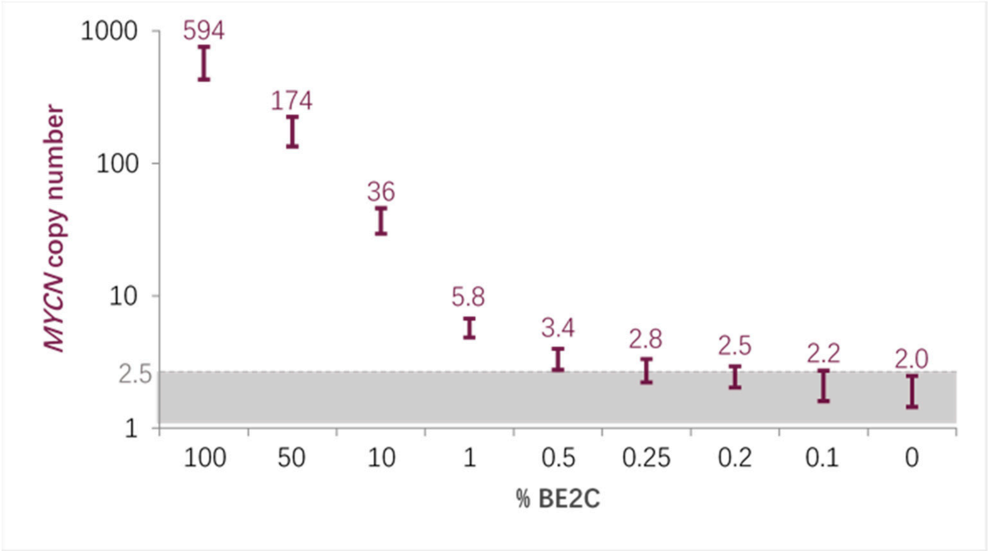

Figure S5. *MYCN* ddPCR copy number limit of detection. BE2C DNA (MNA) titrated with ES8 cell line (diploid *MYCN*) to generate the indicated titration series. Duplex ddPCR copy number assessment of *MYCN* presented with 95% CI. Background shading indicates 2.5 copies threshold as determined by cell line ES8 (0% BE2C).

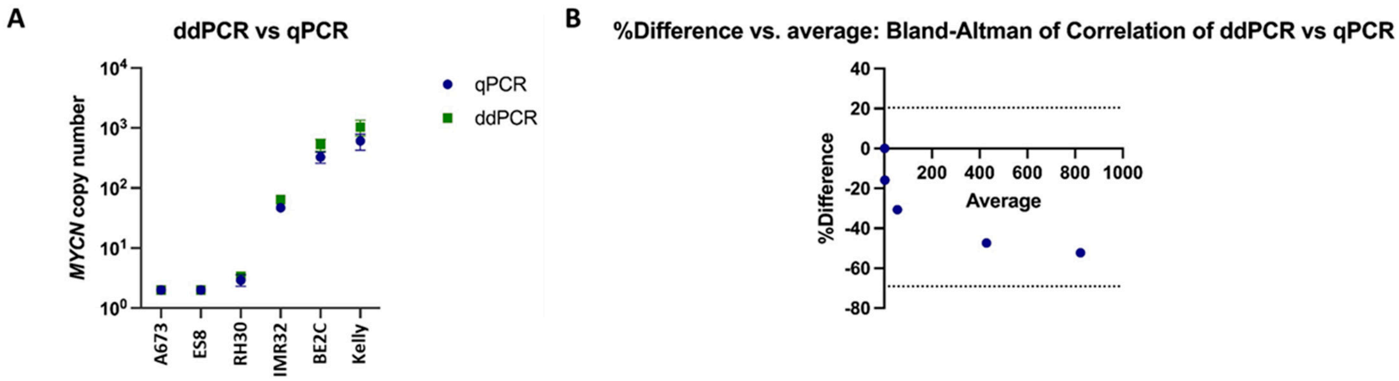

Figure S6. Comparison of MNA data from qPCR and ddPCR. (**A**) Matched duplex ddPCR (green) and duplex qPCR (blue) data for *MYCN* and *THNSL2* copy number, presented with 95% CI. (**B**) Bland and Altman plot for data from (**A**), with the representation of the limits of agreement (dotted lines), from -69.10 to +20.39.

References

1. Monclair, T.; Brodeur, G.M.; Ambros, P.F.; Brisse, H.J.; Cecchetto, G.; Holmes, K.; Kaneko, M.; London, W.B.; Matthay, K.K.; Nuchtern, J.G.; et al. The international neuroblastoma risk group (INRG) staging system: An INRG task force report. *J. Clin. Oncol.* **2009**, *27*, 298–303, doi:10.1200/jco.2008.16.6876.
2. Shimada, H.; Ambros, I.M.; Dehner, L.P.; Hata, J.I.; Joshi, V.V.; Roald, B.; Stram, D.O.; Gerbing, R.B.; Lukens, J.N.; Matthay, K.K.; et al. The International Neuroblastoma Pathology Classification (the Shimada system). *Cancer* **1999**, *86*, 364.
